# Effect of advanced intercrossing on genome structure and on the power to detect linked quantitative trait loci in a multi-parent population: a simulation study in rice

**DOI:** 10.1186/1471-2156-15-50

**Published:** 2014-04-27

**Authors:** Eiji Yamamoto, Hiroyoshi Iwata, Takanari Tanabata, Ritsuko Mizobuchi, Jun-ichi Yonemaru, Toshio Yamamoto, Masahiro Yano

**Affiliations:** 1 National Institute of Agrobiological Sciences, 2-1-2 Kannondai, Tsukuba, Ibaraki 305-8602, Japan; 2 Present address: NARO Institute of Vegetable and Tea Science, National Agriculture and Food Research Organization, 360 Kusawa, Ano, Tsu, Mie 514-2392, Japan; 3 Graduate School of Agricultural and Life Sciences, The University of Tokyo, 1-1-1 Yayoi, Bunkyo, Tokyo 113-8657, Japan; 4 Gene Discovery Research Group, RIKEN Center for Sustainable Resource Science, 3-1-1, Tsukuba, Ibaraki 305-0074, Japan; 5 National Institute of Agrobiological Sciences, 1-2 Ohwashi, Tsukuba, Ibaraki 305-8634, Japan; 6 Present address: NARO Institute of Crop Science, National Agriculture and Food Research Organization, 2-1-18 Kannondai, Tsukuba, Ibaraki 305-8518, Japan

**Keywords:** QTL, Rice, Simulation, Advanced intercrossing

## Abstract

**Background:**

In genetic analysis of agronomic traits, quantitative trait loci (QTLs) that control the same phenotype are often closely linked. Furthermore, many QTLs are localized in specific genomic regions (QTL clusters) that include naturally occurring allelic variations in different genes. Therefore, linkage among QTLs may complicate the detection of each individual QTL. This problem can be resolved by using populations that include many potential recombination sites. Recently, multi-parent populations have been developed and used for QTL analysis. However, their efficiency for detection of linked QTLs has not received attention. By using information on rice, we simulated the construction of a multi-parent population followed by cycles of recurrent crossing and inbreeding, and we investigated the resulting genome structure and its usefulness for detecting linked QTLs as a function of the number of cycles of recurrent crossing.

**Results:**

The number of non-recombinant genome segments increased linearly with an increasing number of cycles. The mean and median lengths of the non-recombinant genome segments decreased dramatically during the first five to six cycles, then decreased more slowly during subsequent cycles. Without recurrent crossing, we found that there is a risk of missing QTLs that are linked in a repulsion phase, and a risk of identifying linked QTLs in a coupling phase as a single QTL, even when the population was derived from eight parental lines. In our simulation results, using fewer than two cycles of recurrent crossing produced results that differed little from the results with zero cycles, whereas using more than six cycles dramatically improved the power under most of the conditions that we simulated.

**Conclusion:**

Our results indicated that even with a population derived from eight parental lines, fewer than two cycles of crossing does not improve the power to detect linked QTLs. However, using six cycles dramatically improved the power, suggesting that advanced intercrossing can help to resolve the problems that result from linkage among QTLs.

## Background

Most agronomically and economically important traits in plants vary quantitatively, and phenotypes of these traits are generally controlled by a combination of many genetic and environmental factors. Naturally occurring genetic variation is a valuable source of alleles for agronomically and economically important traits. In plants, most **q**uantitative **t**rait **l**oci (QTLs) have been identified by using a biparental population such as the F_2_ generation and **r**ecombinant **i**nbred **l**ines (RILs). However, the disadvantage of a biparental population is the reduction in genetic heterogeneity compared with the total genetic variation available for a species. Only two allelic variations are analyzed (one per parent) in a biparental population, which means that useful naturally occurring alleles from other parents might be missed. Another frequently used method for QTL analysis is the association study [1-5]. This strategy uses a large set of varieties and sometimes their wild relatives as a genetic analysis population, and analyzes the association between phenotypes and marker genotypes. The advantage of this strategy is that an association study can detect many naturally occurring allelic variations simultaneously in a single study. However, the application of this strategy in plants is often disturbed by a number of false associations that arise mainly from a highly structured population [5-7]. Nested association mapping (NAM) was designed to combine the advantages of linkage analysis with those of an association study [6,8]. In one use of the NAM strategy, 25 diverse maize inbred lines were crossed with single common inbred line to create 200 RILs for each cross. This produced a total of 5000 RILs that could be used simultaneously in the study. Compared to ordinary association studies, the NAM strategy is less sensitive to the existence of a population structure. An additional advantage of the NAM strategy is that the historical linkage disequilibrium information that is preserved in the parental genomes enables precise mapping of QTLs.

The use of a multi-parent population for QTL analysis has many advantages: accurate specification of the parental origin of alleles [9-14], improvement of mapping resolution by taking advantage of both historical and synthetic recombination, and the use of abundant genetic diversity without the effect of a population structure. The idea of using multi-parent populations in QTL analysis is quite advanced in animal genetics. Heterogeneous stocks in the mouse and in *Drosophila* have been created by means of repeated crosses between eight parental lines over many generations to produce highly recombinant populations [12,15]. The Collaborative Cross is a mouse population derived from eight parent lines followed by inbreeding [16,17]; this material required only one-time genotyping and now enables experiments with the same population in different environments. In plants, inbred lines derived from multiple parents are generally termed **m**ulti-parent **a**dvanced **g**eneration **i**nter-**c**ross (MAGIC) populations [18]. In *Arabidopsis*, a MAGIC population was derived from 19 founder strains followed by four generations of random mating and six generations of selfing [19]. In wheat, a MAGIC population was constructed by inbreeding of four-way F_1_-like progenies [20]. Rice MAGIC populations have been derived from eight parental lines, and two different strategies were applied for their construction [21]. The first strategy used inbreeding of eight-way F_1_-like progenies. The second strategy added two generations of random mating before the inbreeding, and this strategy was termed “MAGIC plus”.

Mapping of QTLs for agronomic traits has revealed that QTLs controlling the same phenotype are often closely linked [22-27]. When two linked QTLs act in opposite directions, it is likely to be difficult to detect them with a population that has relatively few recombination sites, such as an F_2_ population or biparental RILs. Furthermore, in rice, many QTLs tend to be co-localized in specific genomic regions, forming what are known as QTL clusters [28], and these clusters harbor naturally occurring allelic variations of different genes [29]. Because QTL clusters often harbor QTLs related to heading date that affect many other traits, such as culm length and grain yield, this complicates the detection of other QTLs within the same QTL cluster. In both cases, the problems result from linkage among the QTLs.

Linkage among QTLs remains an important issue in the genetic analysis of quantitative traits, and several elaborate theoretical methods have been developed and used [30-32]. In addition, simulation studies have been conducted to design an optimal way to separate linked QTLs in biparental populations. Ronin et al. developed an analytical method to evaluate the expected LOD score for linked QTLs [33]. Mayer compared the power to separate QTLs between regression interval mapping and multiple interval mapping, and found that multiple interval mapping tends to be more powerful as compared to regression interval mapping [34]. Kao and Zeng analyzed the effect of adding self- or random-mating crosses, and found that it was easier to separate QTLs of similar size in the repulsion phase [35]. Li et al. analyzed relationships among the power to separate QTLs, the effect size of each QTL, the population size, and the marker density, and found that dense markers were effective when the population size was sufficiently large [36].

The use of populations that include more recombination sites is expected to be an effective way to resolve the problems that result from linkage among QTLs. To construct a population that includes more recombination sites, an intermated recombinant inbred population (IRIP) strategy with multiple parents is effective. This is an extension of the MAGIC plus approach in rice [21] and is basically the same as the cc04 and cc08 Collaborative Cross populations in the mouse [37]. Because artificial crossing requires a large effort, especially in self-pollinating crops such as rice, it is necessary to design an optimal breeding strategy to minimize the cost and time requirements. In the mouse, an elaborate simulation study for multi-parental populations is available [37]. However, it is difficult to apply those results directly to self-pollinating crops such as rice because of differences between outbred animals and self-pollinating crops. For example, the different mating systems result in differences in the inbreeding procedures used for the construction of inbred lines. In addition, differences in the genome structure between inbred lines generated through siblings and through selfing have been reported [9]. Furthermore, although it has been reported that multi-parent populations can improve the mapping resolution of a QTL by including more recombination sites than ordinary biparental populations [19,37], the efficiency of this approach for the detection of linked QTLs has not been analyzed.

In the present study, we attempted to develop a powerful model for rice that accounts for its differences from the mouse by simulating the construction of rice eight-way IRIPs with different numbers of cycles of recurrent crossing. First, we investigated the effect of advanced intercrossing on the genome structure of each IRIP. We then investigated the effect of advanced intercrossing on the detection of simulated closely linked QTLs.

## Methods

### Production of rice IRIPs

**Figure 1:**
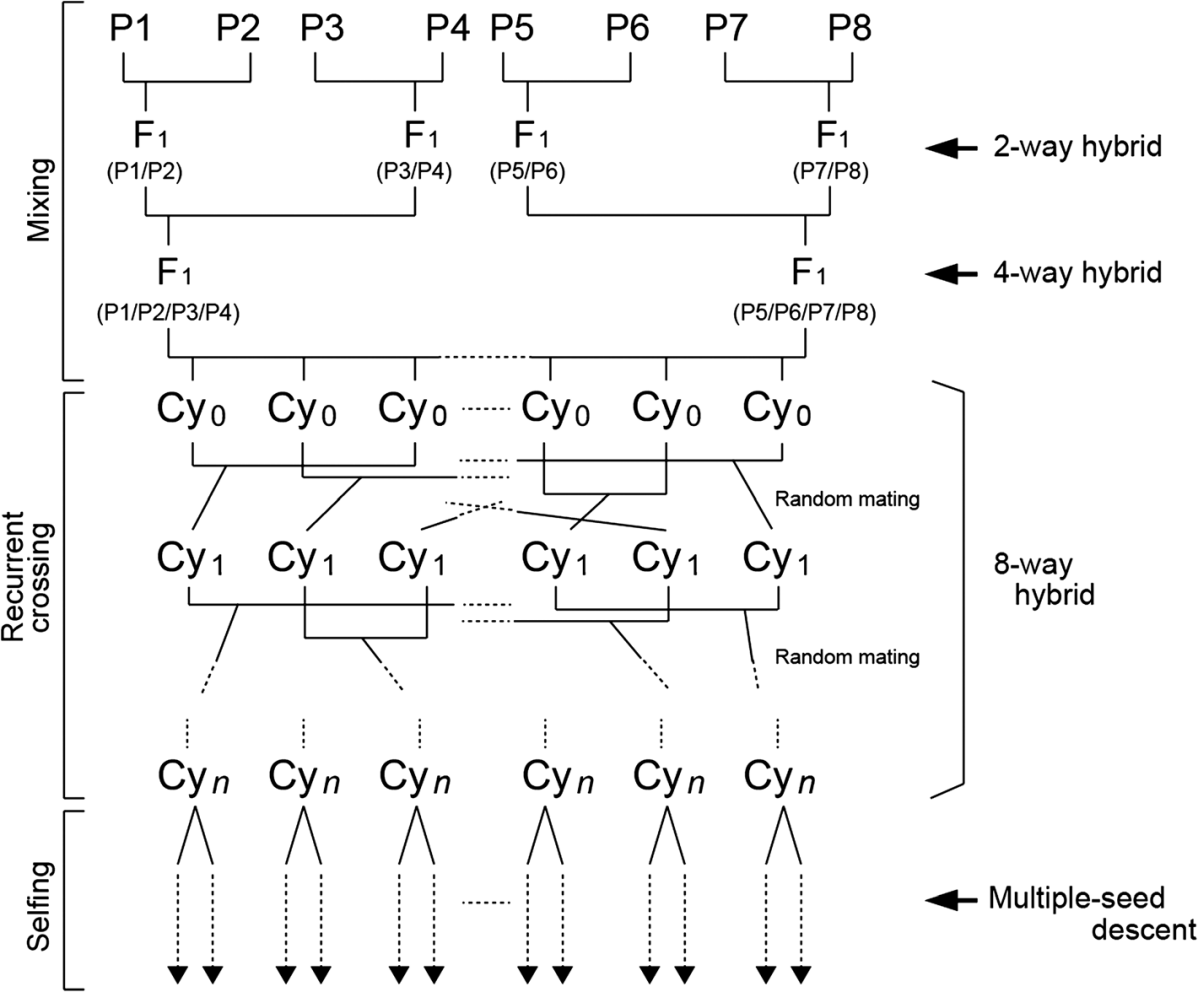
**Strategy used for the production of a rice eight-way IRIP.** Cycles 0 and 1 represent IRIPs derived from no cycles or one cycle of recurrent crossing, respectively. Cy_*n*_, number of cycles.

Because of the successes of eight-way populations [16,17,20,21], we simulated the construction of an eight-way rice IRIP. Figure 1 shows the strategy for the production of the rice IRIP that we used in this study. The strategy is divided into three parts. The first is the mixing stage, in which the genomes of the parental lines are mixed by repeated single crossings. The second is the recurrent crossing stage. This stage is used to increase the number of recombination sites within the population. IRIPs derived from no or two cycles of recurrent crossing (i.e., cycles 0 and 2 in Figure 1) during this stage are the same as the corresponding populations in the rice MAGIC and MAGIC plus designs, respectively [21]. We used disjoint random mating, and produced two progenies from each mating combination in the next generation. Thus, the population size remained constant throughout this stage. The last part of the process is the selfing stage. In this stage, the genomes were genetically fixed by means of repeated inbreeding. To expand the size of the segregating population, we used multiple-seed descent in the first generation of this stage. In the second and subsequent generations, we used single-seed descent. We simulated seven generations of inbreeding, which is expected to fix more than 99% of the genome as homozygous genotypes.

To provide a comparison with the eight-way IRIPs, we also simulated the construction of two-way IRIPs. The strategy is basically the same as the strategy with eight-way IRIPs, but the two-way IRIP does not include a mixing stage.

### Genome structure

The rice genome in this study was represented by the genetic map and chromosome lengths (Table 1) from Harushima et al. [38], with a bin size of 0.1 cM. Thus, we avoided complexities that would result from the existence of recombination hot spots and cold spots at certain physical positions by conducting simulations based on the linkage map positions. The number of crossovers on each chromosome was determined using a random variable drawn from a Poisson distribution. For each chromosome, the lambda parameter of the Poisson distribution (i.e., the expected value of the random variable) was set as the length of the genetic map (in cM) estimated by Harushima et al. [38]. The position of each crossover in a chromosome was sampled from a uniform distribution.

**Table 1:** Rice chromosomal lengths used in the simulations

**Chromosome**	**Length (cM)**
1	181.8
2	157.9
3	166.4
4	129.6
5	122.3
6	124.4
7	118.6
8	121.1
9	93.5
10	83.8
11	117.9
12	109.5

From Harushima et al. [38].

Changes in genome structure were evaluated in terms of the number and length of the genome segments. Non-recombinant genome segments were defined as successive genomic regions composed of only one of the parental genomes.

### QTL conditions

Because most of the QTLs that have been studied in rice have been explained by additive effects only, we assumed that all QTLs in this simulation had only additive effects; that is, we assumed that the dominance and epistasis effects were zero. For all of the settings, the QTL and a marker were considered to be in complete linkage (i.e., co-located at the same position in the chromosome).

QTL conditions for mapping of a single additive QTL are summarized in Table 2. To investigate the mapping accuracy of a single additive QTL, we placed a QTL at the 90-cM position in chromosome 1 (i.e., the middle of the largest chromosome in rice). We defined the mapping accuracy of a single additive QTL as the displacement between the true QTL position and the *M*_1_ position (defined in the section “Power to detect QTLs”).

**Table 2:** QTL conditions for the simulation of power to detect single QTL

		**Allele frequency**	**Additive effect size**								**Location**	
			**P1**	**P2**	**P3**	**P4**	**P5**	**P6**	**P7**	**P8**	**Chr**	**cM**
Figure 4A		4:4	*a*	*a*	*a*	*a*	0	0	0	0	1	90
			*Values assigned to *a* are indicated on *x*-axis of Figure 4A.									
Figure 4B	PVE = 0	4:4	0	0	0	0	0	0	0	0	1	90
		1:7	0	0	0	0	0	0	0	0	1	90
		3:2:3	0	0	0	0	0	0	0	0	1	90
		2:4:2	0	0	0	0	0	0	0	0	1	90
		2:2:2:2	0	0	0	0	0	0	0	0	1	90
		1:1:1:1:1:1:1:1	0	0	0	0	0	0	0	0	1	90
	PVE = 0.02	4:4	0.28	0.28	0.28	0.28	0	0	0	0	1	90
		1:7	0.43	0	0	0	0	0	0	0	1	90
		3:2:3	0.33	0.33	0.33	0.16	0.16	0	0	0	1	90
		2:4:2	0.4	0.4	0.2	0.2	0.2	0.2	0	0	1	90
		2:2:2:2	0.38	0.38	0.26	0.26	0.13	0.13	0	0	1	90
		1:1:1:1:1:1:1:1	0.43	0.37	0.31	0.25	0.19	0.12	0.06	0	1	90
	PVE = 0.04	4:4	0.41	0.41	0.41	0.41	0	0	0	0	1	90
		1:7	0.62	0	0	0	0	0	0	0	1	90
		3:2:3	0.47	0.47	0.47	0.24	0.24	0	0	0	1	90
		2:4:2	0.58	0.58	0.29	0.29	0.29	0.29	0	0	1	90
		2:2:2:2	0.55	0.55	0.36	0.36	0.18	0.18	0	0	1	90
		1:1:1:1:1:1:1:1	0.62	0.53	0.45	0.36	0.27	0.18	0.09	0	1	90
	PVE = 0.06	4:4	0.5	0.5	0.5	0.5	0	0	0	0	1	90
		1:7	0.76	0	0	0	0	0	0	0	1	90
		3:2:3	0.58	0.58	0.58	0.29	0.29	0	0	0	1	90
		2:4:2	0.71	0.71	0.36	0.36	0.36	0.36	0	0	1	90
		2:2:2:2	0.68	0.68	0.45	0.45	0.23	0.23	0	0	1	90
		1:1:1:1:1:1:1:1	0.77	0.66	0.55	0.44	0.33	0.22	0.11	0	1	90
Figure 4C	8-way AF 1/2	4:4	*a*	*a*	*a*	*a*	0	0	0	0	1	90
	8-way AF 1/8	1:7	*a*	0	0	0	0	0	0	0	1	90
	2-way n = 800	1:1	*a*	0	-	-	-	-	-	-	1	90
	2-way n = 200	1:1	*a*	0	-	-	-	-	-	-	1	90
			*Values assigned to *a* are indicated on *x*-axis of Figure 4C.									
Figure 4D	8-way AF 1/2	4:4	0.53	0.53	0.53	0.53	0	0	0	0	1	90
	8-way AF 1/8	1:7	0.53	0	0	0	0	0	0	0	1	90
	2-way n = 800	1:1	0.53	0	-	-	-	-	-	-	1	90
	2-way n = 200	1:1	0.53	0	-	-	-	-	-	-	1	90

QTL conditions for the investigation of the power to detect linked QTLs are summarized in Table 3. For the linked QTLs, we examined two cases. The first case assumes that the additive effects of the two linked QTLs act in opposite directions (i.e., the QTLs are in the repulsion phase; Table 3). In this case, we placed two QTLs with the same effect size but with the effects acting in opposite directions. In the second case, we assumed that the additive effects of two linked QTLs were both positive (QTLs in coupling phases; Table 3). In this case, we placed two QTLs that both had positive additive effects. In both cases, QTL1 was placed at the 90-cM position in chromosome 1 and QTL2 was placed at the position 90 + *x* cM position in chromosome 1, where *x* was set to 5, 10, or 20 cM. The distribution of a QTL allele among the parents affects the probability of recombination between two linked QTLs during the mixing stage (Figure 1). Therefore, we prepared two conditions for the distribution of the QTL allele among the parents. In the first, the alleles from parents P1, P3, P5, and P7 possess the effect of the QTL and alleles from the other parents have no effect on the phenotype. We describe this arrangement of alleles as the “highest frequency” arrangement (Table 3). In the second, the alleles from parents P1, P2, P3, and P4 possess the effect of the QTL and alleles from the other parents have no effect on the phenotype. We describe this arrangement of alleles as the “lowest frequency” arrangement (Table 3). In this experiment, the environmental noise was set to be *N* (0, 1). Therefore, PVE of the simulated QTLs is different from each other. Distributions of actual PVE in this experiment are indicated in Additional files 1 and 2.

**Table 3:** QTL conditions for the simulation of power to detect linked QTLs

			**Additive effect size**								**Location**	
			**P1**	**P2**	**P3**	**P4**	**P5**	**P6**	**P7**	**P8**	**Chr**	**cM**
Figure 5A, B (Repulsion)	Highest	QTL1	0.53	0.00	0.53	0.00	0.53	0.00	0.53	0.00	1	90
		QTL2	-0.53	0.00	-0.53	0.00	-0.53	0.00	-0.53	0.00	1	90 + *x*
	Lowest	QTL1	0.53	0.53	0.53	0.53	0.00	0.00	0.00	0.00	1	90
		QTL2	-0.53	-0.53	-0.53	-0.53	0.00	0.00	0.00	0.00	1	90 + *x*
Figure 5C (Coupling)	Highest	QTL1	0.53	0.00	0.53	0.00	0.53	0.00	0.53	0.00	1	90
		QTL2	0.53	0.00	0.53	0.00	0.53	0.00	0.53	0.00	1	90 + *x*
	Lowest	QTL1	0.53	0.53	0.53	0.53	0.00	0.00	0.00	0.00	1	90
		QTL2	0.53	0.53	0.53	0.53	0.00	0.00	0.00	0.00	1	90 + *x*
Figure 5D, Table 6 (Coupling of small QTLs)	Highest	QTL1	0.27	0.00	0.27	0.00	0.27	0.00	0.27	0.00	1	90
		QTL2	0.27	0.00	0.27	0.00	0.27	0.00	0.27	0.00	1	90 + *x*
	Lowest	QTL1	0.27	0.27	0.27	0.27	0.00	0.00	0.00	0.00	1	90
		QTL2	0.27	0.27	0.27	0.27	0.00	0.00	0.00	0.00	1	90 + *x*
Figure 5E (Repulsion)	4:4	QTL1	0.53	0.53	0.53	0.53	0.00	0.00	0.00	0.00	1	90
		QTL2	-0.53	-0.53	-0.53	-0.53	0.00	0.00	0.00	0.00	1	100
	3:2:3	QTL1	0.61	0.61	0.61	0.30	0.30	0.00	0.00	0.00	1	90
		QTL2	-0.61	-0.61	-0.61	-0.30	-0.30	0.00	0.00	0.00	1	100
	2:4:2	QTL1	0.75	0.75	0.37	0.37	0.37	0.37	0.00	0.00	1	90
		QTL2	-0.75	-0.75	-0.37	-0.37	-0.37	-0.37	0.00	0.00	1	100
	2:2:2:2	QTL1	0.71	0.71	0.47	0.47	0.24	0.24	0.00	0.00	1	90
		QTL2	-0.71	-0.71	-0.47	-0.47	-0.24	-0.24	0.00	0.00	1	100
	1:1:1:1:1:1:1:1	QTL1	0.81	0.69	0.58	0.46	0.35	0.23	0.12	0.00	1	90
		QTL2	-0.81	-0.69	-0.58	-0.46	-0.35	-0.23	-0.12	0.00	1	100
Figure 5F (Coupling)	4:4	QTL1	0.53	0.53	0.53	0.53	0.00	0.00	0.00	0.00	1	90
		QTL2	0.53	0.53	0.53	0.53	0.00	0.00	0.00	0.00	1	100
	3:2:3	QTL1	0.61	0.61	0.61	0.30	0.30	0.00	0.00	0.00	1	90
		QTL2	0.61	0.61	0.61	0.30	0.30	0.00	0.00	0.00	1	100
	2:4:2	QTL1	0.75	0.75	0.37	0.37	0.37	0.37	0.00	0.00	1	90
		QTL2	0.75	0.75	0.37	0.37	0.37	0.37	0.00	0.00	1	100
	2:2:2:2	QTL1	0.71	0.71	0.47	0.47	0.24	0.24	0.00	0.00	1	90
		QTL2	0.71	0.71	0.47	0.47	0.24	0.24	0.00	0.00	1	100
	1:1:1:1:1:1:1:1	QTL1	0.81	0.69	0.58	0.46	0.35	0.23	0.12	0.00	1	90
		QTL2	0.81	0.69	0.58	0.46	0.35	0.23	0.12	0.00	1	100
Figure 6A (Repulsion)	8-way AF 1/2	QTL1	0.53	0.53	0.53	0.53	0.00	0.00	0.00	0.00	1	90
		QTL2	-0.53	-0.53	-0.53	-0.53	0.00	0.00	0.00	0.00	1	100
	8-way AF 1/8	QTL1	0.53	0.00	0.00	0.00	0.00	0.00	0.00	0.00	1	90
		QTL2	-0.53	0.00	0.00	0.00	0.00	0.00	0.00	0.00	1	100
	2-way	QTL1	0.53	0.00	-	-	-	-	-	-	1	90
		QTL2	-0.53	0.00	-	-	-	-	-	-	1	100
Figure 6B (Coupling)	8-way AF 1/2	QTL1	0.53	0.53	0.53	0.53	0.00	0.00	0.00	0.00	1	90
		QTL2	0.53	0.53	0.53	0.53	0.00	0.00	0.00	0.00	1	100
	8-way AF 1/8	QTL1									1	90
		QTL2	0.53	0.00	0.00	0.00	0.00	0.00	0.00	0.00	1	100
	2-way	QTL1	0.53	0.00	-	-	-	-	-	-	1	90
		QTL2	-0.53	0.00	-	-	-	-	-	-	1	100

Environmental noise was determined to be *N* (0,1) in all conditions.

Values assigned to *x* are indicated in caption of corresponding figures.

In this study, we compared *n* = 800 in the eight-way population with *n* = 200 and 800 in the two-way population. We determined the size of a two-way population with *n* = 200 using the following logic: First, given that eight parental lines were chosen and that we tried to use all of the available genetic diversity in these parents, the resulting eight-way population is analogous to four two-way populations with no replication of the parental lines. If the size of each two-way population is *n* = 200, the sum of the sizes of the four populations is four times this size (i.e., *n* = 4 × 200 = 800), which is the same size as the eight-way population that we simulated.

We also simulated the power to detect multiple QTLs. Effect size and allele frequency of each QTL was selected from conditions described in Table 4 according to the following rules. In Experiment 1, we based the distribution of 11 loci and their chromosomal locations on the known positions of rice blast resistance QTLs (Table 5). In general, the QTLs for blast resistance can be divided into two patterns: either the QTL is multi-allelic and each variety possesses an allele with a different level of effect, or the QTL is bi-allelic and only one or a limited number of varieties possesses the allele with measurable effects. Therefore, in this experiment, we assumed that the distribution of four loci and their allelic distribution follow allele frequency “4:4” in Table 4, whereas another four loci follow “1:1:1:1:1:1:1:1”. Allelic distributions of the remaining three loci were determined randomly. Among the eleven loci, one locus was selected from variance of additive effects of a QTL 0.03 in Table 4, five loci from 0.04, three loci from 0.05, and two loci from 0.06. Combination of allele frequency and QTL variance were determined randomly in each simulation. In Experiment 2, we included nine loci whose chromosomal locations were based on the positions of known heading date QTLs (Table 5). Many heading date QTLs are bi-allelic, though several are multi-allelic. Therefore, we assumed the following distribution of these QTLs: two loci per condition followed “4:4”, “2:6”, and “1:7”, and one locus per model followed “3:2:3”, “2:4:2”, and “2:2:2:2” (Table 4). Among the nine loci, two loci were selected from variance of additive effects of a QTL 0.04 in Table 4, two loci from 0.05, three loci from 0.06, and two loci from 0.07. Experiment 3 includes ten QTLs whose chromosomal locations were based on known QTLs for seed morphology (Table 5). Because QTLs for seed morphology are often bi-allelic and correspond to the population structure in rice (i.e., the allelic pattern can be divided into *indica* or *japonica*, the two main sub-species in cultivated rice), we defined the allelic distribution of QTLs for the eight loci using “4:4” and the distribution for the remaining two loci using a randomly determined condition (Table 4). Among the ten loci, two loci were selected from variance of additive effects of a QTL 0.04 in Table 4, six loci from 0.05, and two loci from 0.06. Environmental noise was determined to be *N* (0, 0.5) in all simulations. Thus, our simulation conditions were stochastic (i.e., based on actual positions of known QTLs, but with random assignment of their effect). Distributions of actual PVE in this experiment are indicated in Additional file 3.

**Table 4:** QTL conditions for the simulation of multiple-QTLs

**Allele frequency**	**Variance of the additive effects of a QTL**	**List of additive effect size**							
4:4	0.03	0.35	0.35	0.35	0.35	0.00	0.00	0.00	0.00
	0.04	0.40	0.40	0.40	0.40	0.00	0.00	0.00	0.00
	0.05	0.45	0.45	0.45	0.45	0.00	0.00	0.00	0.00
	0.06	0.49	0.49	0.49	0.49	0.00	0.00	0.00	0.00
	0.07	0.53	0.53	0.53	0.53	0.00	0.00	0.00	0.00
2:6	0.03	0.40	0.40	0.00	0.00	0.00	0.00	0.00	0.00
	0.04	0.46	0.46	0.00	0.00	0.00	0.00	0.00	0.00
	0.05	0.52	0.52	0.00	0.00	0.00	0.00	0.00	0.00
	0.06	0.57	0.57	0.00	0.00	0.00	0.00	0.00	0.00
	0.07	0.61	0.61	0.00	0.00	0.00	0.00	0.00	0.00
1:7	0.03	0.52	0.00	0.00	0.00	0.00	0.00	0.00	0.00
	0.04	0.60	0.00	0.00	0.00	0.00	0.00	0.00	0.00
	0.05	0.68	0.00	0.00	0.00	0.00	0.00	0.00	0.00
	0.06	0.74	0.00	0.00	0.00	0.00	0.00	0.00	0.00
	0.07	0.80	0.00	0.00	0.00	0.00	0.00	0.00	0.00
3:2:3	0.03	0.40	0.40	0.40	0.20	0.20	0.00	0.00	0.00
	0.04	0.46	0.46	0.46	0.23	0.23	0.00	0.00	0.00
	0.05	0.52	0.52	0.52	0.26	0.26	0.00	0.00	0.00
	0.06	0.57	0.57	0.57	0.28	0.28	0.00	0.00	0.00
	0.07	0.61	0.61	0.61	0.31	0.31	0.00	0.00	0.00
2:4:2	0.03	0.49	0.49	0.24	0.24	0.24	0.24	0.00	0.00
	0.04	0.57	0.57	0.28	0.28	0.28	0.28	0.00	0.00
	0.05	0.63	0.63	0.32	0.32	0.32	0.32	0.00	0.00
	0.06	0.69	0.69	0.35	0.35	0.35	0.35	0.00	0.00
	0.07	0.75	0.75	0.37	0.37	0.37	0.37	0.00	0.00
2:2:2:2	0.03	0.46	0.46	0.31	0.31	0.15	0.15	0.00	0.00
	0.04	0.54	0.54	0.36	0.36	0.18	0.18	0.00	0.00
	0.05	0.60	0.60	0.40	0.40	0.20	0.20	0.00	0.00
	0.06	0.66	0.66	0.44	0.44	0.22	0.22	0.00	0.00
	0.07	0.71	0.71	0.47	0.47	0.24	0.24	0.00	0.00
1:1:1:1:1:1:1:1	0.03	0.53	0.45	0.38	0.30	0.23	0.15	0.08	0.00
	0.04	0.61	0.52	0.44	0.35	0.26	0.17	0.09	0.00
	0.05	0.68	0.59	0.49	0.39	0.29	0.20	0.10	0.00
	0.06	0.75	0.64	0.53	0.43	0.32	0.21	0.11	0.00
	0.07	0.81	0.69	0.58	0.46	0.35	0.23	0.12	0.00

**Table 5:** Chromosomal (Chr) distribution of the simulated QTLs

**Chr**	**cM**	**Corresponding QTLs**
Experiment 1: Blast resistance		
1	140	*Pi37*
2	156	*Pib*
4	53	*Pi21*
6	56	*Pi9*
6	67	*Pi-d2*
8	22	*Pi36*
9	32	*Pi5-1*
11	33	*Pia*
11	91	*Pb1*
11	117	*Pik1*
12	50	*Pi-ta*
Experiment 2: Heading date		
3	6	*dth3*
3	144	*Hd6*
6	10	*Hd17*
6	12	*Hd3a*
6	51	*Hd1*
6	59	*DTH2*
7	50	*Ghd7*
8	35	*Ghd8*
10	43	*Ehd1*
Experiment 3: Seed morphology		
1	87	*Rd*
2	37	*GW2*
3	83	*GS3*
3	102	*qGL3*
4	59	*GIF1*
5	28	*GS5*
5	36	*qSW5*
6	91	*TGW6*
7	43	*qSD7-1/qPC7*
8	106	*qGW8*

### Power to detect QTLs

For QTL mapping, we distributed markers with eight polymorphisms at 1-cM intervals throughout the rice genome. This marker condition set is far from the currently available marker sets, but we will provide a justification for this approach in the Discussion. Using the *F*-test, we detected a significant association between marker genotypes and the phenotypes observed in the segregating population. There are several elaborate methods that enable the separation of linked QTLs [30-32]. However, as described above, we assumed a simple situation for our simulation. The aim of this study was to investigate the potential of an eight-way IRIP to resolve problems derived from linkage among QTLs, not to compare the performance of various theoretical methods. To simplify our simulation and make it computationally feasible, we used the following strategy to detect linked QTLs, which is similar to the strategy used in the scantwo function of R/qtl [39]. In the QTL analysis, we considered the following two models:

$$H_{2}:y=\mu +\beta_{1}q_{1}+\beta_{2}q_{2}+\epsilon$$

$$H_{1}:y=\mu +\beta_{1}q_{1}+\epsilon$$

where *H*_2_ and *H*_1_ are the two-QTL and single-QTL models, respectively; μ represents the population mean, β_*x*_ represents the additive effect of QTL_*x*_, *q*_*x*_ represents the coded variable for the QTL genotype of QTL_*x*_, and ϵ represents the residual error. As we noted earlier, we did not account for epistasis or dominance effects in the models. We then defined three indices for detecting QTLs:

$$M_{2}=\max_{c\left(s\right)=i,c\left(t\right)=j}\left\{-\log_{10}P_{\left(s,t\right)}\right\}$$

$$M_{1}=\max_{c\left(s\right)=i\;\mathrm{or}\;j}\left\{-\log_{10}P_{s\;}\right\}$$

$$M_{2\mathrm{vs}1}=M_{2}-M_{1}$$

where *i* and *j* indicate the chromosome number, including the case when *i* = *j*, and *c* (*s*) and *c* (*t*) denote the chromosomes for loci *s* and *t*, respectively. *P*_*s*_ is the *P*-value from the *F*-test at locus *s*, and *P*_(*s, t*)_ is the *P*-value from loci *s* and *t* (*s* ≠ *t*). *M*_2_ indicates the fit of the two-QTL model, and was used in the experiments for separating two linked QTLs. *M*_1_ indicates the fit of the single-QTL model, and was used in all experiments in this study. *M*_2vs1_ indicates whether the two-QTL model provides a sufficiently improved fit over the best single-QTL model to justify its use. To investigate the power of an eight-way IRIP to separate linked QTLs, we used the following rule:

$$M_{2}>T_{2}\;\text{and}\;M_{2\mathrm{vs}1}>T_{2\mathrm{vs}1}$$

where *T*_2_ and *T*_2vs1_ indicate genome-wide significance thresholds for *M*_2_ and *M*_2vs1_, respectively.

Although genome-wide significance thresholds can be obtained by means of a permutation test, this approach is computationally infeasible in our case because of the large number of simulations required. In the present study, we determined the genome-wide significance thresholds following the method of Valdar et al. [37]. First, we simulated a null distribution for *M*_1_, *M*_2_, and *M*_2vs1_ by repeating 10 000 simulations with only environmental noise included. In the null simulations, a low number of repeats often results in underestimation of the significance thresholds, and it has been suggested that estimating thresholds by using a generalized extreme-value model is more efficient than taking empirical quantiles [37]. Therefore, we fit a generalized extreme value by means of the maximum-likelihood method to the values obtained from the null simulations using the “evd” package of the R software [40]. We chose the 95th percentile of the null distribution as the significance threshold for each experimental condition (Table 6). In this study, we defined detection of a QTL when the values of *M*_1_, *M*_2_, and *M*_2vs1_ within 20 cM from the true position of the QTL or QTLs exceeded the genome-wide significance threshold (Table 6). That is, for mapping of a single QTL, *M*_1_ was obtained in the range from 70 to 110 cM on chromosome 1. In the case of mapping of two QTLs, *M*_1_, *M*_2_, and *M*_2vs1_ were obtained in the range from 70 to (110 + *x*) cM, where *x* is 5, 10 or 20 cM. In other words, we defined significant signals in other genomic regions as false positives because their chromosomal locations were too far from the true positions of the simulated QTLs.

**Table 6:** Estimated 5% genome-wide significance threshold from 10 000 null simulations

		**Number of cycles**									
		**0**	**1**	**2**	**4**	**6**	**8**	**10**	**12**	**20**	**22**
8-way IRIPs (*n* = 800)	*T*_1_	4.03	4.03	4.09	4.2	4.11	4.1	4.22	-	4.23	-
	*T*_2_	4.74	4.81	4.86	4.95	4.97	4.87	5.03	-	5.19	-
	*T*_2vs1_	2.45	2.47	2.34	2.3	2.23	2.16	2.26	-	2.32	-
2-way IRIPs (*n* = 200)	*T*_1_	4.09	-	4.02	4.15	4	4.11	4.15	4.19	-	4.15
	*T*_2_	4.99	-	5.17	5.25	5.2	5.28	5.38	5.43	-	5.43
	*T*_2vs1_	2.98	-	2.93	2.69	2.67	2.51	2.44	2.4	-	2.23
2-way IRIPs (*n* = 800)	*T*_1_	4.00	-	3.90	3.82	3.86	3.67	4.22	4.02	-	4.10
	*T*_2_	4.61	-	4.44	4.46	4.73	4.22	5.21	4.92	-	4.75
	*T*_2vs1_	2.82	-	2.81	2.73	2.44	2.18	2.66	2.42	-	2.06

*T*_1_ represents the thresholds for the single-QTL model. *T*_2_ represents the thresholds for the two-QTL model, and *T*_2vs1_ represents the thresholds if whether the two-QTL model provides a sufficiently improved fit over the best single-QTL model to justify its use.

## Results

### Effect of genetic drift during the recurrent crossing stage

**Figure 2:**
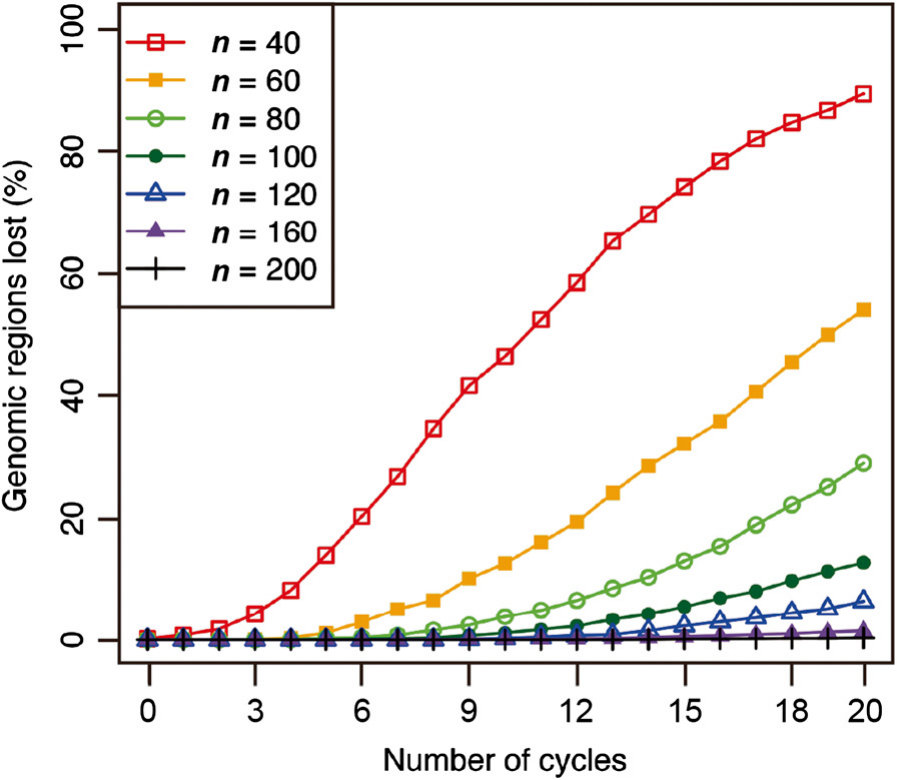
**Frequency of genetic drift during the recurrent crossing stage.** The degree of genetic drift was represented by the percentage of the total genomic regions in which the genome derived from one or more of the parental lines had been lost. *n* represents the population size.

In the construction of an IRIP, it is preferable to use a larger population size during the recurrent crossing stage (Figure 1) to create a larger number of recombination sites within the population [41]. However, a huge number of crosses are an unrealistic goal, especially in a self-pollinating crop, and a smaller population size is preferable for actual breeding operations. On the other hand, a small population will suffer from the effects of genetic drift, which will result in the loss of some parental genomic regions from the population. As the first step of this study, we therefore simulated the relationship between population size during the recurrent crossing stage and the effect of genetic drift to see if we could find an optimal solution. We measured the degree of genetic drift as a percentage of the total genomic regions where genomes derived from one or more of the parental lines had been lost (i.e., where the number of marker alleles in the population was less than eight). As we expected, a small population size increased the percentage of genomic regions affected by genetic drift as the number of cycles increased, and a larger population size decreased the frequency of lost regions (Figure 2). At a population size of *n* = 100, the proportion of the genomic regions affected by genetic drift remained less than 1% until 10 cycles of recurrent crossing and was about 10% even after 20 cycles (Figure 2). Because we thought this magnitude of genetic drift was acceptably small and the population size was at a realistic level for actual operations, we adopted a population size of *n* = 100 for our subsequent simulations. We also tested *n* = 200 for some simulations, but because the results were similar to those with *n* = 100, we have not shown the data.

### Relationships between the number of recurrent crossings and the genome structure

**Figure 3:**
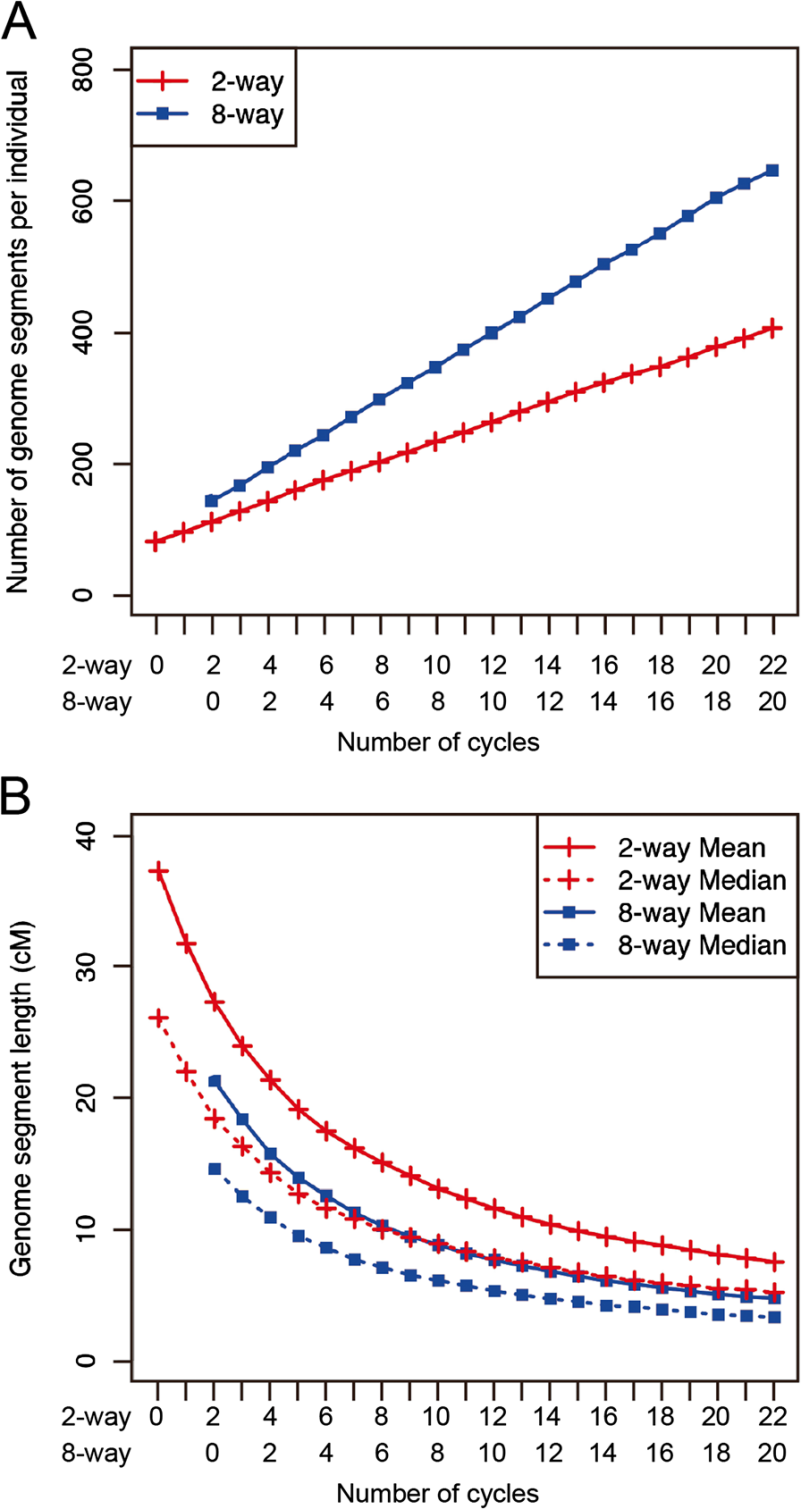
**Relationship between the number of cycles and the genome structure in a rice two-way IRIP (*n*** **= 200) and an eight-way IRIP (*n*** **= 800).** Plots for the eight-way IRIP started two cycles behind the two-way IRIP to match the total number of outcrossings (i.e., the eight-way population requires two additional outcrossings to reach the cycle 0 stage). **(A)** Total number of genome segments per individual. **(B)** Mean and median genome segment lengths.

We evaluated the effect of recurrent crossing on the genome structure of individuals in an IRIP in terms of the number and length of the genome segments. The number of genome segments per individual increased with increasing number of cycles during the recurrent crossing stage (Figure 3A). In contrast, the length of the genome segments was inversely related to the number of cycles (Figure 3B). The mean and median genome segment lengths both decreased dramatically during the first five to six cycles, but decreased more slowly during subsequent cycles (Figure 3B). We also investigated the differences in the genome structure between the two-way and eight-way IRIPs (Figure 3). The difference between the two-way and eight-way IRIPs in the number of genome segments increased as the number of cycles increased (Figure 3A); however, the difference in the length of these segments decreased as the number of cycles increased (Figure 3B). The mean and median genome segment lengths were higher than those observed in the mouse Collaborative Cross. For example, in cycle 4 for the eight-way IRIP, mean genome segment lengths were 8.6 and 13.9 cM in the mouse [37] and rice (Figure 3B) crosses, respectively. This is probably due to the different inbreeding strategy; that is, the mouse strategy used siblings and the rice strategy used selfing to construct the inbred lines.

### Power to detect QTLs in rice eight-way IRIPs

**Figure 4:**
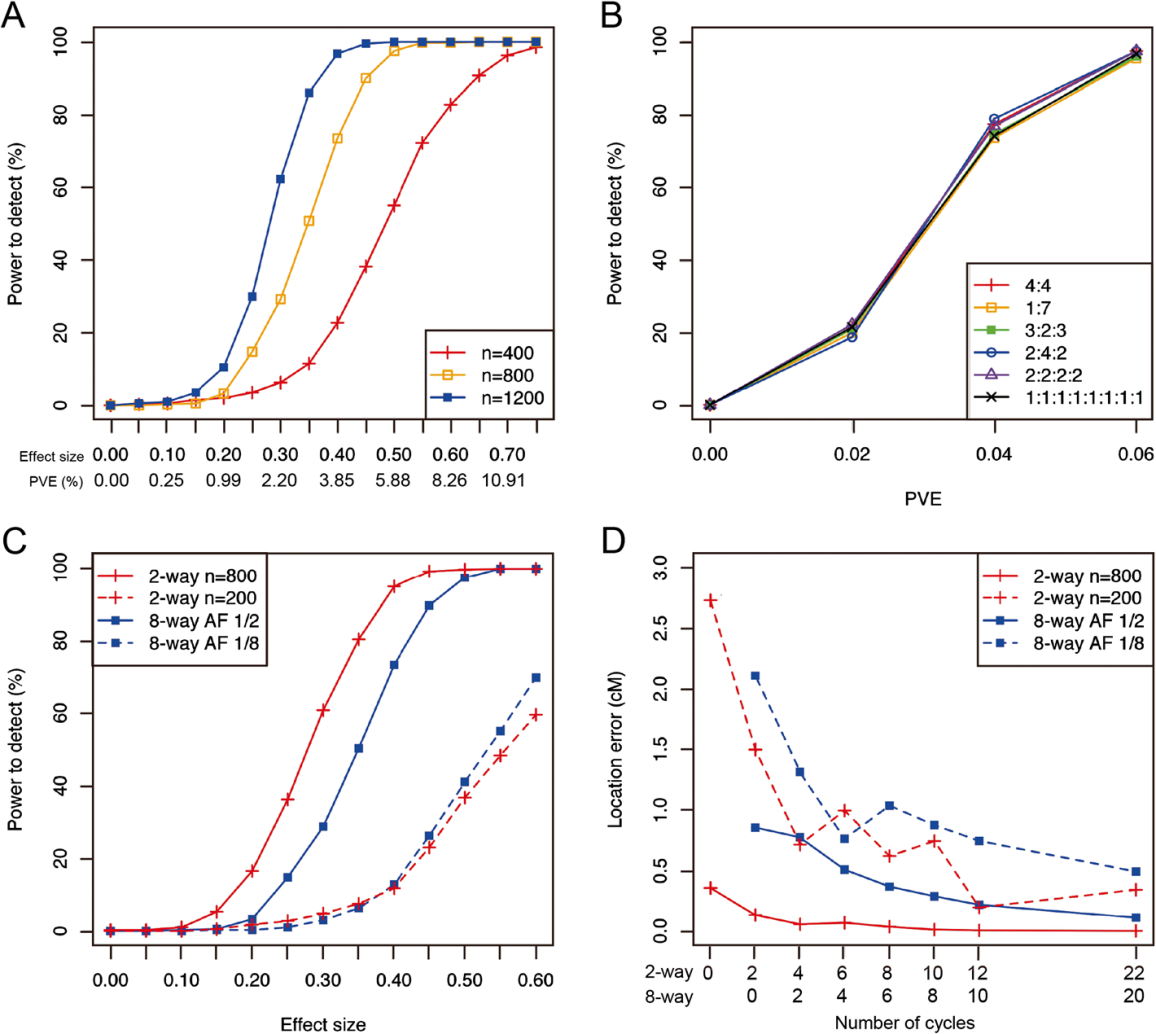
**Power to detect single additive QTLs in the rice eight-way IRIPs.** Detailed conditions of this experiment are described in Table 2. **(A)** Power to detect a single additive QTL in the rice eight-way IRIPs. Values are the result of 400 simulations. **(B)** Comparison of the power to detect a single additive QTL between the bi-allelic case and the multi-allelic cases. Values are the result of 400 simulations. **(C)** Comparison of the power to detect a single additive QTL between the two-way and eight-way IRIPs. Values are the result of 400 simulations. **(D)** The location error for the detected QTLs. The location error equals the distance between the position of the maximum *P*-value and the true QTL position. Values are the result of 1000 simulations.

The detection of a QTL generally depends on the population size, allele frequency, and size of the effect. The two latter factors determine the PVE that is more indicative for the power to detect. Therefore, for the simulation of power to detect a single additive QTL, we described both the effect size and the corresponding PVE (Figure 4A). The detection power was saturated at PVE values of 0.120, 0.065, and 0.045 when *n* = 400, 800, and 1200, respectively (Figure 4A). These results agree well with the simulation results in the mouse Collaborative Cross [37]. In multi-parent populations, segregating QTLs are expected to be multi-allelic. We also compared the power to detect between the bi-allelic and multi-allelic cases (Table 2; Figure 4B). It should be noted that the same PVE value at a different allele frequency indicates a different size of the additive effect (Table 2). If the QTL possessed the same PVE value in both cases, then the number of alleles for the QTL had little effect on the power to detect the QTL (Figure 4B).

It would be interesting to compare the relative power of the two-way and eight-way IRIP designs. However, this is a difficult challenge because of differences in the total phenotypic variance. In general, an eight-way population includes more segregating QTLs, and this results in a larger genetic variance that leads to a larger total phenotypic variance. This changes the PVE of a QTL with the same effect size and therefore changes the power to detect the QTL. Because the change in the total phenotypic variance depends on the parental lines used to create the study population, it is difficult to estimate. In the following simulations, we assumed a simple situation in which only one QTL is involved in the phenotype and the environmental noise is constant (i.e., *N* (0, 1)). This may be unrealistic, but it provides a good preliminary estimate of the QTL’s characteristics because it is easy to interpret the results obtained by the simulations.

In comparing the two-way and eight-way populations, the reduction of the frequency of the QTL alleles should also be considered. In the two-way population, the QTL allele frequency is always 1/2, whereas it ranges from a minimum of 1/8 to a maximum of 1/2 in the eight-way population. Therefore, for the eight-way population, we simulated two cases: one in which the QTL allele frequency is 1/2, and another in which the frequency is 1/8 (Table 2 and Figure 4C). When the allele frequency of the QTL was 1/2 in the parental lines, the power to detect was higher in the large eight-way population (*n* = 800) than in the smaller two-way population (*n* = 200; Figure 4C). When the allele frequency of the QTL was 1/8 in the parental lines, the power to detect was similar in the two populations (Figure 4C). However, it should be noted that we did not consider the increase in the total phenotypic variance in the eight-way population as described above, and therefore, the detection power in the eight-way population is only an estimate.

We also investigated the location error of the detected QTLs (Figure 4D). Despite large differences in the genome structure between the rice IRIP (Figure 3B) and the mouse Collaborative Cross population [37], little difference was observed in mapping accuracy (Figure 4D, [37]). Our comparison of the location error between the two-way and eight-way IRIPs provided results similar to those for the power to detect. That is, when the allele frequency of the QTL was 1/2 in the parental lines, the location error in the large eight-way IRIP (*n* = 800) was smaller than that in the smaller two-way IRIP (*n* = 200), but when the allele frequency of the QTL was 1/8 in the parental lines, the location error was similar in both IRIPs (Figure 4D).

**Figure 5:**
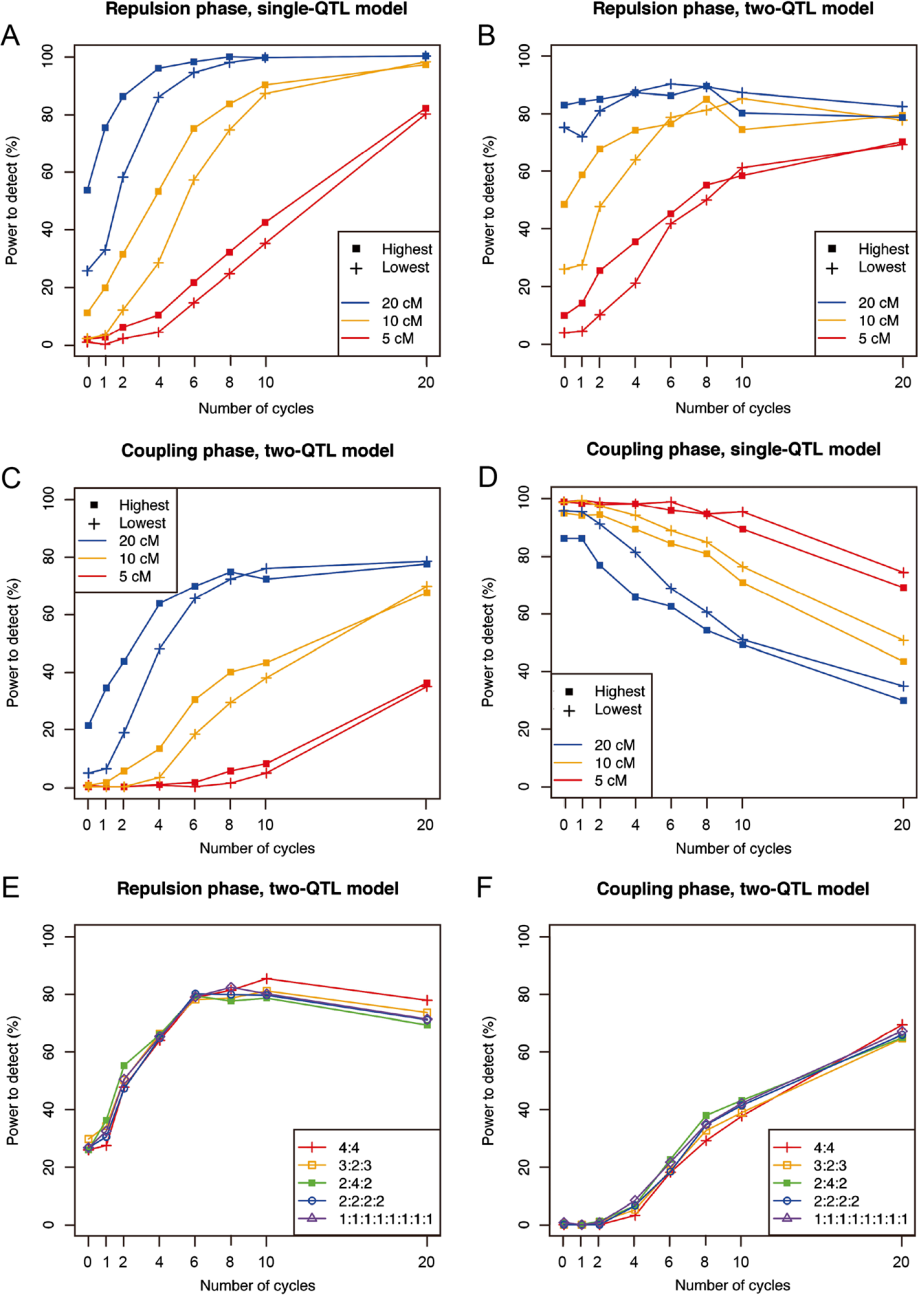
**Relationship between the number of cycles and the power to detect linked QTLs.** Data are the results for the rice eight-way IRIPs (*n* = 800). Values are the result of 400 simulations, and 5, 10, and 20 cM represent the distance between the QTLs. Detailed conditions of this experiment are described in Table 3. **(A)** Power to detect QTLs in the repulsion phase by using the single-QTL model. **(B)** Power to detect QTLs in the repulsion phase by using the two-QTL model. **(C)** Power to separate QTLs in the coupling phase by using the two-QTL model. **(D)** Power to detect QTLs with a small effect in the coupling phase. **(E)** Comparison of the power to separate QTLs in the repulsion phase between the bi-allelic and multi-allelic cases. **(F)** Comparison of the power to separate QTLs in the coupling phase between the bi-allelic and multi-allelic cases.

We then simulated the power to separate linked QTLs in eight-way IRIPs. First, we simulated the case where the additive effects of two linked QTLs act in opposite directions (i.e., QTLs in the repulsion phase; Table 3). In this case, QTLs cannot be detected if there is insufficient recombination between the QTLs because their alleles have opposite effects and negate each other’s effects. Therefore, an increased number of cycles will be required to increase the power to detect QTLs. First, we investigated the detection power by using the single-QTL model. The detection power under this simulation setting was indicated by the relative power compared with the case in which the QTLs are unlinked. As expected, an increased number of cycles improved the power to detect QTLs in the repulsion phase (Figure 5A, B). It was interesting that even when the distance between QTLs in the repulsion phase was 20 cM, which is larger than the size of most of QTL clusters [28], the power to detect linked QTLs was less than 50% of that in the case with unlinked QTLs after zero cycles, but increased rapidly with an increasing number of cycles (Figure 5A). By using the two-QTL model, the detection power improved dramatically compared with the results using the single-QTL model (Figure 5B). However, if the QTL interval was 5 cM, the power was less than 50% until four cycles (Figure 5B). Another important result is that using fewer than two cycles showed little improvement compared to using zero cycles (Figure 5B).

When the additive effects of the linked QTLs are both large and positive (i.e., QTLs in coupling phase; Table 3), they are often mistakenly estimated as a single QTL with a large effect at the wrong position. Therefore, we also investigated the effectiveness of the IRIP approach to separate two linked QTLs in the coupling phase by using the two-QTL model. In general, it is more difficult to separate two QTLs in the coupling phase than in the repulsion phase [35]. Our results confirmed this problem (Figure 5C). To achieve more than 50% detection power for the separation required more than ten cycles when the QTL interval was 10 cM (Figure 5C). When the QTL interval was 5 cM, it required more than 20 cycles to achieve 50% detection power (Figure 5C). As in the case of QTLs in the repulsion phase, using fewer than two cycles showed little improvement compared to using zero cycles (Figure 5C). In addition, we simulated the power to detect QTLs in the coupling phase in the following situation: If two linked QTLs are closely linked, the *P*-value obtained by using the single-QTL model is sufficiently large to achieve statistical significance because such QTLs behave as if they are a single QTL. However, if the linkage between two QTLs is broken by means of repeated crossing, those QTLs become undetectable because the effect size of each QTL is too small to achieve statistical significance (Coupling of small QTLs; Table 3). First, we simulated the detection of such QTLs by using the single-QTL model. As expected, the detection power decreased as the number of cycles increased (Figure 5D). In the analysis, using the two-QTL model gave a power near 0% in all simulated cases, even though the two-QTL model fit the completely correct model for these simulated data (Table 7). In addition, we investigated the differences in power to separate linked QTLs between the bi-allelic and multi-allelic cases (Figure 5E, F). The number of alleles for the QTL had little effect on the power to separate (Figure 5E, F).

**Table 7:** Power to detect QTLs with a small effect that are linked in the coupling phase

	**Highest frequency**			**Lowest frequency**		
	**5 cM**	**10 cM**	**20 cM**	**5 cM**	**10 cM**	**20 cM**
Cycle 0	0.0	0.0	0.2	0.0	0.0	0.3
Cycle 1	0.0	0.2	0.4	0.3	0.0	0.3
Cycle 2	0.0	0.4	0.5	0.0	0.0	0.0
Cycle 6	0.0	0.4	2.0	0.4	0.2	0.5
Cycle 10	0.5	0.7	1.9	0.3	1.3	1.9

Experimental conditions for these results are summarized in Table 3, using the two-QTL model. Values are the result of 400 simulations.

**Figure 6:**
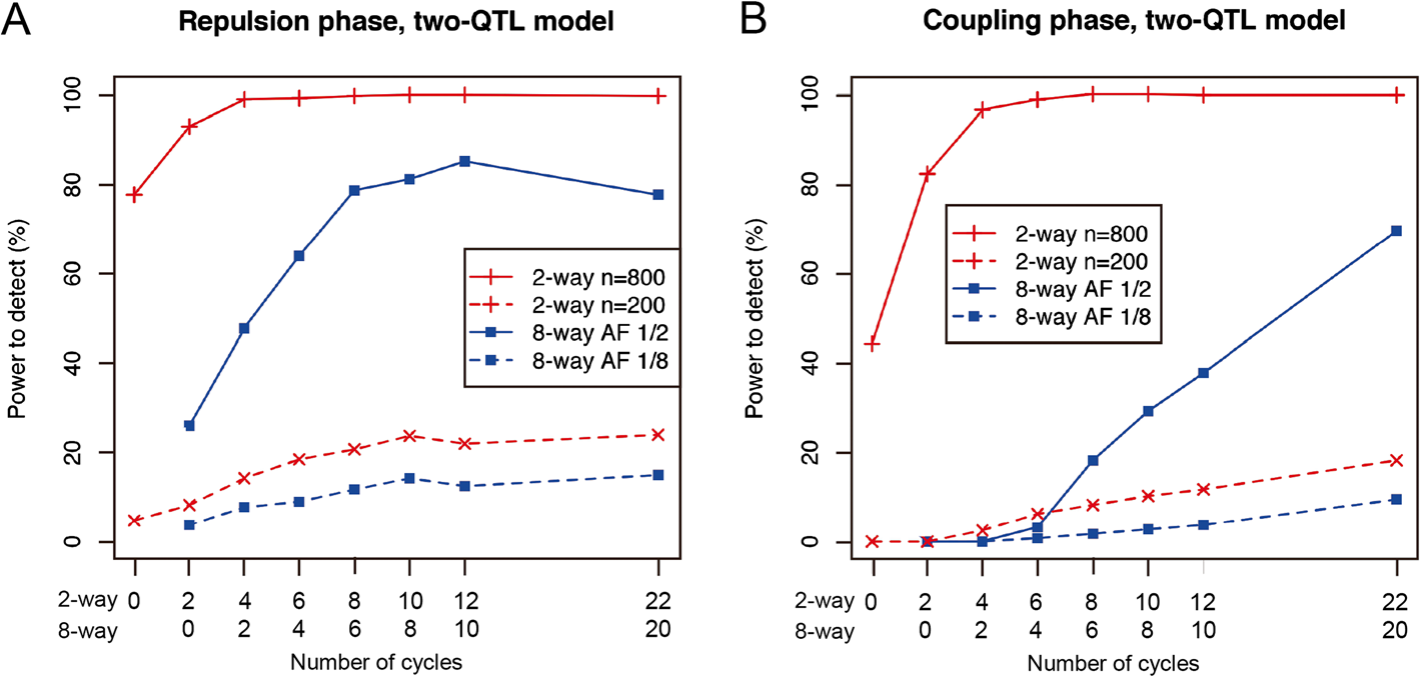
**Comparison of the power to separate linked QTLs between the two-way and eight-way IRIPs.** Plots for the eight-way population started two cycles behind the two-way IRIP to match the number of outcrossings (i.e., the eight-way population requires two additional outcrossings to reach the cycle 0 stage). Values are the result of 400 simulations. Detailed conditions of this experiment are described in Table 3. **(A)** Power to detect QTLs in the repulsion phase by using the two-QTL model. **(B)** Power to separate QTLs in the coupling phase by using the two-QTL model.

We also simulated the power to separate two QTLs between the two-way and eight-way IRIPs (Figure 6). The results resembled those for the power to detect a single QTL (Figure 4B). That is, when the allele frequency of the QTL was 1/2 in the parental lines, the power to detect was higher in the large eight-way IRIP (*n* = 800) than in the smaller two-way IRIP (*n* = 200), but when the allele frequency of the QTL was 1/8 in the parental lines of the eight-way IRIP, the power to detect was similar in both IRIPs (Figure 6).

### Power to detect multiple QTLs

In the previously described simulations, we assumed the segregation of only one or two target QTLs and assigned the rest of the variance to environmental noise (Tables 2 and 3). These simple situations enabled us to interpret the results more easily. However, in general, many QTLs with different effect sizes and a different number of alleles segregate simultaneously in populations. To investigate the effectiveness of multi-parent IRIPs in an actual QTL mapping study in rice, we simulated the power to detect multiple QTLs by using three different experiments (Tables 4 and 5). In this simulation, we compared the power between an eight-way population with *n* = 800 and four two-way populations with *n* = 200. In the latter case, we defined the detection of QTLs as a situation in which at least one of the two-way populations produced a significant signal in the target region. For all three experiments that we simulated, the eight-way population detected more QTLs than in the four two-way populations (Table 8). Because all the experiments included two combinations of closely linked QTLs (i.e., QTLs on the same chromosome in Table 5), increasing the number of cycles is expected to improve the power to detect the QTLs, as shown in Figures 5 and 6. The effectiveness of increasing the number of cycles was larger in the eight-way population than in the two-way population (Table 8).

**Table 8:** Mean of the number of detected QTLs in each experiment

		**Experiment 1**	**Experiment 2**	**Experiment 3**
2-way	Cycle 0	6.35 ± 0.73	5.48 ± 0.63	6.51 ± 0.54
	Cycle 8	6.44 ± 0.65	5.37 ± 0.71	6.45 ± 0.66
8-way	Cycle 0	9.96 ± 0.69	7.63 ± 0.68	8.91 ± 0.72
	Cycle 6	10.68 ± 0.49	8.09 ± 0.64	9.67 ± 0.49
	Total number of QTLs	11	9	10

Experiments are based on the QTL data in Tables 4 and 5.

Values are the mean of 100 simulations.

In the two-way population, we defined detection of the QTL as a situation in which at least one of the four two-way populations produced a significant signal in the target region.

## Discussion

In the present study, we simulated the construction of an eight-way IRIP for rice and examined its power to separate linked QTLs. Because the construction of such populations requires a large effort, especially in self-pollinating crops such as rice, we should carefully determine the optimal design for developing such an IRIP. In this study, we investigated the efficiency of advanced intercrossing for developing rice IRIPs and improving the QTL detection power as a function of the population size and number of cycles of recurrent crossing.

Rice eight-way IRIPs are potentially useful as breeding materials. The idea of using such populations as breeding material resembles the “genome shuffling” that is used in the breeding of microbes [42,43]. Genome shuffling emphasizes that chimeric genes or genomes derived from repeated genomic recombination can improve the performance of the progenies. In addition, rice QTL clusters appear to be composed of different but tightly linked genes [28,29]. Because rice breeding is mainly conducted through the pedigree method, introgressions have often resulted in the replacement of large genome segments that are sufficiently large to include all QTL cluster regions [44]. Given this evidence, lines with a chimeric genome structure within their QTL clusters will be good materials for breeding because they include new combinations of QTLs that are unavailable in current varieties because of tight linkage among QTLs. Rice QTL clusters average about 15 cM in size [28]. In rice eight-way populations, the mean and median genome segment lengths were both more than 15 cM after zero cycles. However, both parameters became less than 15 cM within five or six cycles (Figure 3B). Thus, using five or six cycles appears to be effective based on the results for the whole genome structure and for the structure within a QTL cluster.

In the simulation of the power to detect QTLs, we placed markers with eight polymorphisms at 1-cM intervals throughout the rice genome and used them to estimate the number of recombination sites in the genome. This approach could be implemented using, for example, 1551 simple-sequence-repeat markers with eight polymorphisms or 4653 single-nucleotide-polymorphism markers for which each of three marker sets are tightly linked and constitute haplotype polymorphisms that can distinguish among the eight ancestral genomes at the marker position. Recently, a high-density single-nucleotide-polymorphism genotyping system has been undergoing development [45-49], and highly elaborate statistical methods have been developed to estimate the parental origins [9-14]. Based on this research, our assumption about the marker conditions used in this study seems to be sufficiently realistic. Recent advances in next-generation sequencing technologies have enabled re-sequencing of a large number of genomes [50]. Application of these technologies to IRIP genotyping will enable more accurate mapping of QTLs in rice eight-way IRIPs.

As mentioned above, linkage among QTLs is problematic in rice QTL analysis. Therefore, we investigated the power to detect linked QTLs in a rice eight-way IRIP. If the distance between QTLs in the repulsion phase was 20 cM, the detection power after zero cycles using the single-QTL model was less than 40% of the power for unlinked QTLs (Figure 5A). The distance of 20 cM is larger than the size of most QTL clusters in rice [28]. Thus, even when the population was derived from eight parental lines, using zero cycles of recurrent crossing creates a risk of missing QTLs with a large effect because their alleles in the same phase have opposite effects. Although the power to detect QTLs in the repulsion phase was dramatically improved by using the two-QTL model, this required a combination of the two-QTL model with at least six cycles of recurrent crossing to achieve 50% power to detect QTLs within a 5-cM region (Figure 5B). Moreover, separating QTLs in the coupling phase required more cycles to achieve sufficient improvement in the detection power than would be required in the repulsion phase (Figure 5C). Another important finding is that using fewer than two cycles showed little or no improvement over using zero cycles (Figure 5B, C). Collectively, the simulation results suggest that several cycles of recurrent crossing will be necessary to resolve the problems derived from linkage among QTLs even when the populations are derived from eight parental lines. On the other hand, we also showed that, in some cases, linked QTLs with a small additive effect size can be detected only in a population with fewer recombination sites (Figure 5D). This result was caused by overestimation of a single QTL’s effect by failing to separate two QTLs, thus the information obtained is incorrect in a precise sense. However, QTL mapping projects are initiated for a variety of purposes, and in an agronomic study, researchers are often interested in obtaining information that will guide future selection experiments. In this case, it is enough to identify a genomic region that affects the target phenotype, even if the obtained information is ambiguous (i.e., if two QTLs in the coupling phase are not separated). Therefore, although we have demonstrated the importance of advanced intercrossing, we also note the merit of using a population produced by zero cycles of recurrent crossing in some cases, especially for agronomic purposes. In general, the construction of inbred lines in self-pollinating crops is easier than in outbreeding species. One method to resolve the trade-off in the number of cycles required may be to construct inbred lines using different numbers of cycles. This method will increase the likelihood of detecting QTLs in both repulsion and coupling phases.

We also compared the power to detect and separate the QTLs between the two- and eight-way IRIPs. Based on the simplifying assumption that only one or two target QTLs were segregating in the populations, the two-way populations had similar or higher power to detect QTLs (Figures 4 and 6). However, in two-way populations, it is possible that both parents have the same allele for the target QTL. In this case, the QTL cannot be detected even if some of the QTL’s alleles have a large effect. In the simulations to detect multiple QTLs (Tables 4 and 5), the eight-way IRIPs had higher detection power than the two-way populations (Table 8). Thus, if the allele frequency or distribution are not known *ab initio*, the eight-way IRIP is a safer alternative despite the risk of decreasing the power to detect and separate QTLs in some cases.

In this study, we simulated the construction of rice eight-way IRIPs and discovered that even with a relatively small number of cycles, recurrent crossing effectively produces a highly recombinant and chimeric genome structure and therefore improves the power to detect QTLs. Although recurrent crossing is effective, its efficiency depends on factors such as the population size and the number of cycles. Because our simulation was performed under a range of conditions, the results will be useful for determining the optimal IRIP design for a given experimental objective. Although we designed our study for application of the IRIP approach to rice, the results can be applied to other crops with similar characteristics (e.g., self-pollinating species in which quantitative genetic studies have been conducted mainly with inbred lines derived from a bi-parental cross).

## Conclusion

In the genetic analysis of agronomic traits, linkage among QTLs can complicate the detection of each individual QTL. By using information for rice, we simulated the construction of an eight-way population followed by cycles of recurrent crossing and inbreeding, and investigated the resulting genome structure and its usefulness for detecting linked QTLs as a function of the number of cycles of recurrent crossing. Our results indicated that even when the population is derived from eight parental lines, the use of fewer than two cycles does not improve the power to detect linked QTLs. However, increasing to six cycles dramatically improved the detection power, suggesting that advanced intercrossing can help to resolve the problems derived from linkage among QTLs.

## Abbreviations

IRIP: Intermated recombinant inbred population; PVE: Proportion of variance explained; QTL: Quantitative trait locus.

## Competing interests

The authors declare that they have no competing interests.

## Authors’ contributions

EY, HI, TT, RM, JY, TY, and MY designed the research. EY, HI, and TT conducted the research. EY and HI wrote the manuscript. All authors read and approved the final manuscript.

## Supplementary Material

Additional file 1 **Distribution of PVEs of the simulated QTLs.** A to E correspond to the distribution of PVEs of the simulated QTLs used in Figure 5A and B, C, D, E and F, respectively. Click here for file

Additional file 2 **Distribution of PVEs of the simulated QTLs.** A and B correspond to the distribution of PVEs of the simulated QTLs used in Figure 6A and B. Click here for file

Additional file 3 **Distribution of PVEs of the simulated QTLs.** Experiment 1 to 3 correspond to those in Table 8. Click here for file
