# Supplementary material for: Effect of advanced intercrossing on genome structure and on the power to detect linked quantitative trait loci in a multi-parent population: a simulation study in rice
Source: BMC Genet. 2014 Apr 27;15:50. doi: 10.1186/1471-2156-15-50 (PMC4101851; doi:10.1186/1471-2156-15-50)

Additional file 1. Distribution of PVEs of the simulated QTLs. A to E correspond to the distribution of PVEs of the simulated QTLs used in Fig 5A and B, C, D, E and F, respectively.

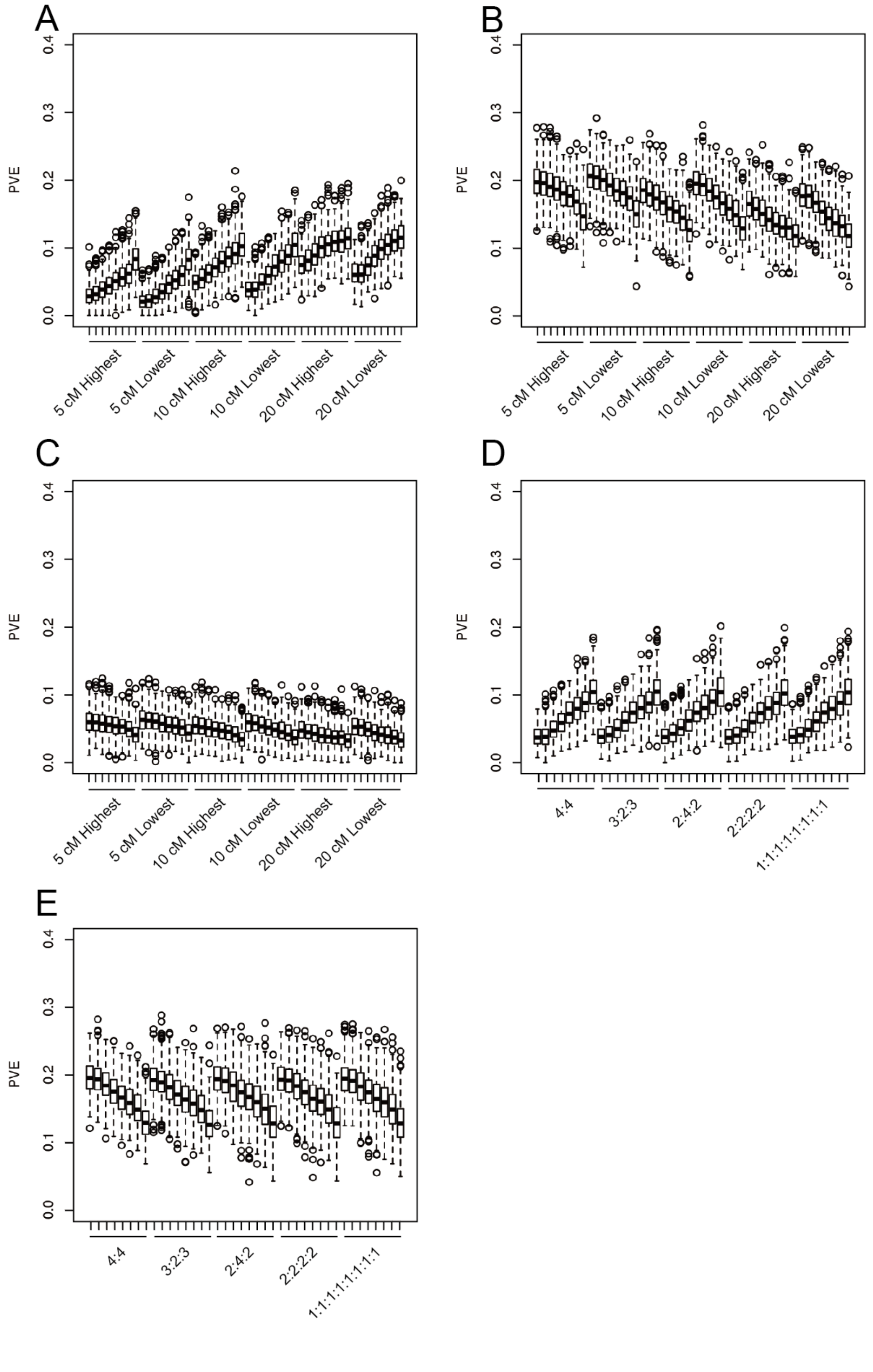

Supplement: Additional file 1 — Distribution of PVEs of the simulated QTLs. A to E correspond to the distribution of PVEs of the simulated QTLs used in Figure 5A and B, C, D, E and F, respectively. [file 1471-2156-15-50-S1.pdf]
