# Supplementary material for: Effect of advanced intercrossing on genome structure and on the power to detect linked quantitative trait loci in a multi-parent population: a simulation study in rice
Source: BMC Genet. 2014 Apr 27;15:50. doi: 10.1186/1471-2156-15-50 (PMC4101851; doi:10.1186/1471-2156-15-50)

Additional file 2. Distribution of PVEs of the simulated QTLs. A and B correspond to the distribution of PVEs of the simulated QTLs used in Fig. 6A and B.

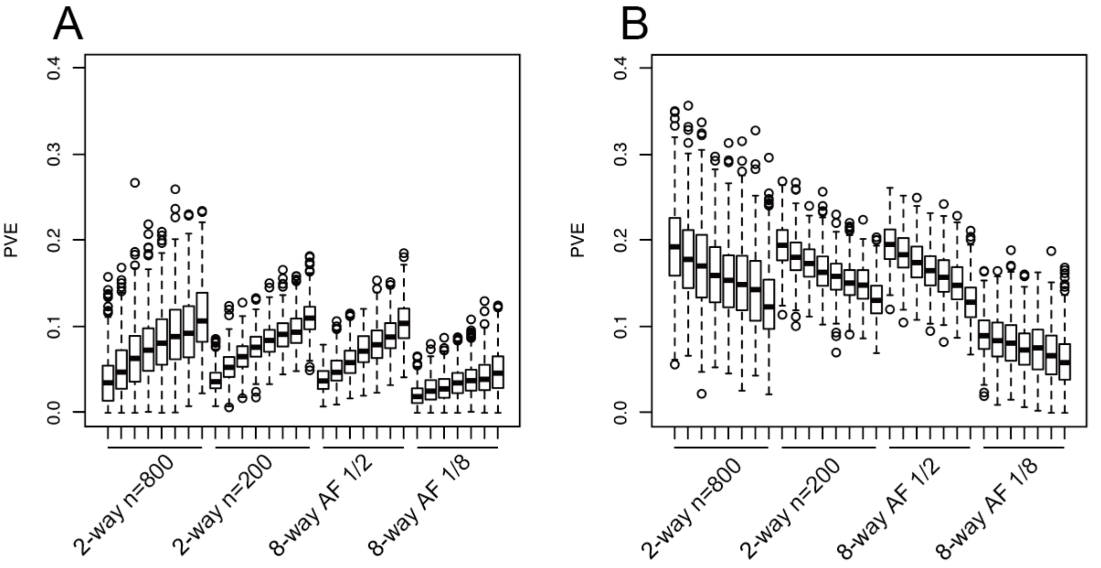

Supplement: Additional file 2 — Distribution of PVEs of the simulated QTLs. A and B correspond to the distribution of PVEs of the simulated QTLs used in Figure 6A and B. [file 1471-2156-15-50-S2.pdf]
