# Supplementary material for: Effect of advanced intercrossing on genome structure and on the power to detect linked quantitative trait loci in a multi-parent population: a simulation study in rice
Source: BMC Genet. 2014 Apr 27;15:50. doi: 10.1186/1471-2156-15-50 (PMC4101851; doi:10.1186/1471-2156-15-50)

Additional file 3. Distribution of PVEs of the simulated QTLs. Experiment 1 to 3 correspond to those in Table 8.

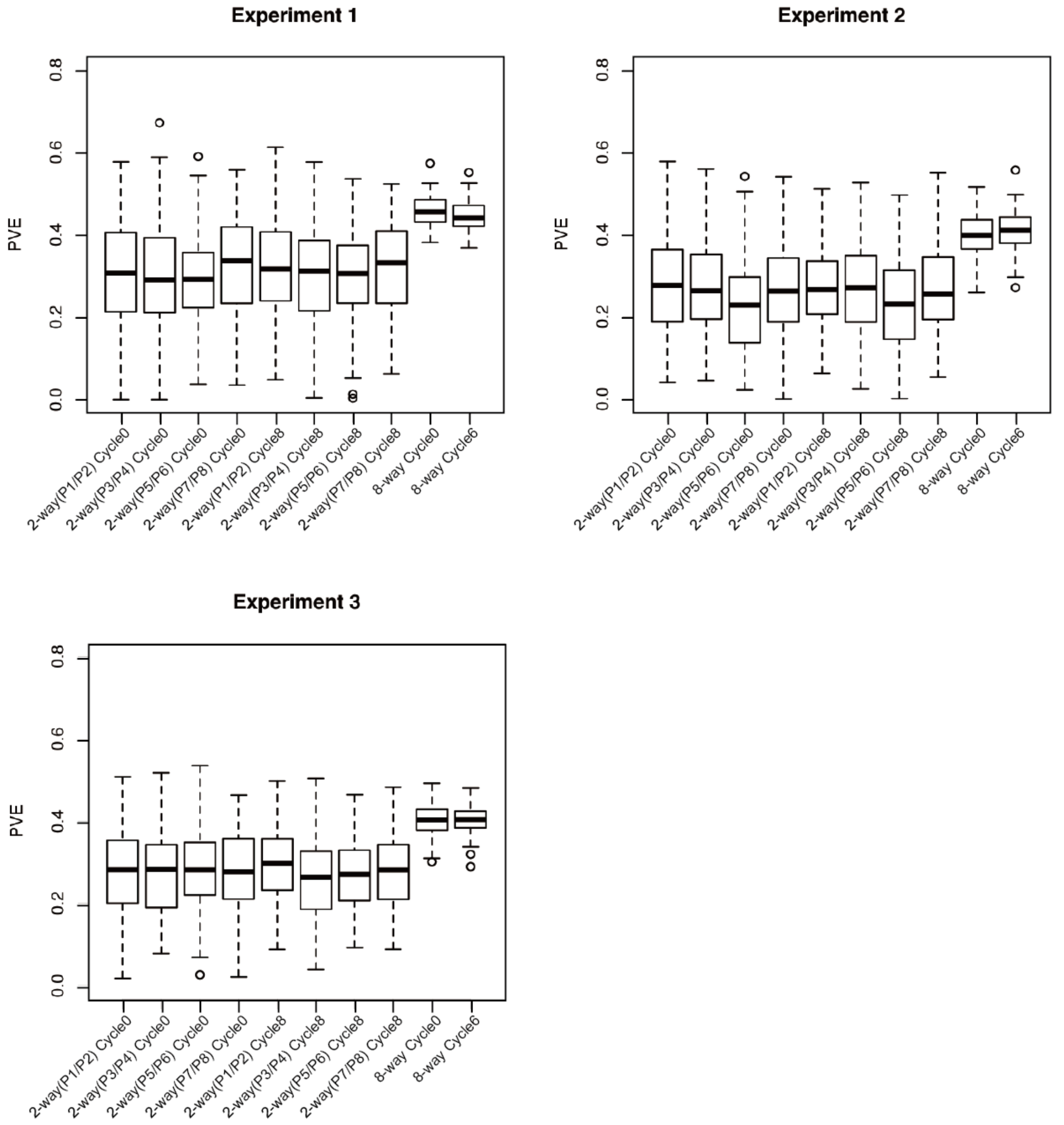

Supplement: Additional file 3 — Distribution of PVEs of the simulated QTLs. Experiment 1 to 3 correspond to those in Table 8. [file 1471-2156-15-50-S3.pdf]
